# Correction: Association of CYP2A6*4 with Susceptibility of Lung Cancer: A Meta-Analysis

**DOI:** 10.1371/annotation/1e88e5e7-a6f9-426b-b9c5-26fc923a23c2

**Published:** 2013-08-26

**Authors:** Lishan Wang

The content of this Correction is superseded by the Retraction [2] and has been updated on June 7, 2022.
